# Linear decomposition approach for a class of nonconvex programming problems

**DOI:** 10.1186/s13660-017-1342-y

**Published:** 2017-04-13

**Authors:** Peiping Shen, Chunfeng Wang

**Affiliations:** 1 College of Mathematics and Information Science, Henan Normal University, Xinxiang, 453007 P.R. China; 2 Henan Engineering Laboratory for Big Data Statistical Analysis and Optimal Control, Henan Normal University, Xinxiang, 453007 P.R. China

**Keywords:** 90C30, 90C33, 90C15, nonconvex programming, global optimization, linear decomposition approach, approximation algorithm, computational complexity

## Abstract

This paper presents a linear decomposition approach for a class of nonconvex programming problems by dividing the input space into polynomially many grids. It shows that under certain assumptions the original problem can be transformed and decomposed into a polynomial number of equivalent linear programming subproblems. Based on solving a series of liner programming subproblems corresponding to those grid points we can obtain the near-optimal solution of the original problem. Compared to existing results in the literature, the proposed algorithm does not require the assumptions of quasi-concavity and differentiability of the objective function, and it differs significantly giving an interesting approach to solving the problem with a reduced running time.

## Introduction

Consider a class of nonconvex programming problems:

$$\text{(P)}:\quad \textstyle\begin{cases} \text{min }f(x)=\varphi(a^{\top}_{1}x,a^{\top}_{2}x,\ldots,a^{\top }_{k}x),\\ \text{s.t. } x\in\Omega=\{x\in\mathbb{R}^{n}|Ax\leq b,x\geq0\}, \end{cases} $$

where $k\geq2$, $\varphi:\mathbb{R}^{k}\rightarrow\mathbb{R}_{+}$ is a continuous function, Ω is a nonempty polytope, $b\in\mathbb {R}^{s}$, $A\in\mathbb{R}^{s\times n}$, and $a_{1},a_{2},\ldots,a_{k}\in \mathbb{R}^{n}$ are linear independent vectors. The function *f* is called a low-rank function with rank *k* over a polytope Ω defined by Kelner and Nikovola [1]. With this broader definition, multiplicative programming, quadratic programming, bilinear programming, as well as polynomial programming can be all put into the category of problem (P), whose important applications can be found in some surveys (e.g., [2–7]). In general, nonconvex programming problems of this form (P) are known to be NP-hard, even minimizing the product of two linear functions with rank two over a polytope is NP-hard ([8]). As shown by Mittal and Schulz [9], the optimum value of problem (P) cannot be approximated to within any factor unless $\mathrm{P}=\mathrm{NP}$. Hence, for solving problem (P) some extra assumptions ($\mathbb{A}1$)-($\mathbb {A}3$) on the properties of the function *f* will be required as follows:

($\mathbb{A}1$)
$\varphi(y)\leq\varphi(y^{\prime})$, if $y_{i}\leq y_{i}^{\prime}$, for each $i=1,\ldots,k$;
($\mathbb{A}2$)
$\varphi(\lambda y)\leq\lambda^{c}\varphi(y)$ for all $y\in\mathbb{R}_{+}^{k},\lambda>1$ and some constant *c*;
($\mathbb{A}3$)
$a^{\top}_{i}x>0,\text{ for } i=1,\ldots,k$.

An exhaustive reference on optimizing low-rank functions can be found in Konno and Thach [10]. Konno et al. [11] proposed cutting plane and tabu-search algorithms for low-rank concave quadratic programming problems. Porembski [12] gave a cutting plane solution approach for general low-rank concave minimization problems with a small number of variables. Additionally, some solution algorithms have been developed for the special cases of problem (P) (e.g. [13–16]). The above solution methods are efficient heuristics, without providing a theoretical analysis on the running time or performance of the algorithms.

The main purpose of this article is to present an approximation scheme with provable performance bounds for solving globally problem (P) to obtain an *ε*-approximate solution for any $\varepsilon>0$ in time polynomial in the input size and $\frac{1}{\varepsilon}$. For the special cases of problem (P), there exists extensive work about the solution of *ε*-approximation problems. Vavasis [17] gave an approximation scheme for low-rank quadratic optimization problems. Depetrini and Locatelli [18] presented a fully polynomial-time approximation scheme (FPTAS) for minimizing the sum or product of ratios of linear functions over a polyhedron. Kelner and Nikolova [1] developed an expected polynomial-time smoothed algorithm for a class of low-rank quasi-concave minimization problems whose the objective function satisfies the Lipschitz condition. Daniele and Locatelli [19] proposed an FPTAS for minimizing product of two linear functions over a polyhedral set. Additionally, for minimizing the product of two non-negative linear cost functions, Goyal et al. [20] gave an FPTAS under the condition of the convex hull of the feasible solutions in terms of linear inequalities known. The algorithm in [21] works for minimizing a class of low-rank quasi-concave functions over a convex set, and this algorithm solves a polynomial number of linear optimization problems. Mittal and Schulz [9] presented an FPTAS for minimizing a general class of low-rank functions over a polytope, and their algorithm is based on constructing an approximate Pareto-optimal front of the linear functions that constitute the objective function.

In this paper, by exploiting the feature of problem (P), a suitable nonuniform grid for solving problem (P) is first constructed over a given $(k-1)$-dimensional box. Based on the exploration of the grid nodes, the original problem (P) can then be transformed and decomposed into a polynomial number of subproblems, in which each subproblem is corresponding to a grid node and is easy to solve by considering a linear program. Thus, the main computational effort of the proposed algorithm only consists in solving linear programming problems related to all nodes, which do not grow in size from a grid node to the next node. Furthermore, it is verified that through solving these linear programs, we can obtain an *ε*-approximation solution of the primal problem (P). The proposed algorithm has several features as follows. First, in contrast with [19, 20, 22], the rank *k* of the objective function considered by the proposed algorithm is not limited to only around two. Second, the proposed algorithm does not require differentiable and the inverse of the single variable function about the objective function, and it works for minimizing a class of more general functions, while Goyal and Ravi [21] and Kelner and Nikolova [1] both require the quasi-concavity assumption of the objective function. Third, although the nonuniform grid constructed for the algorithms in [21] and ours is based on subdividing a $(k-1)$-dimensional hyper-rectangle, the algorithm in [21] requires iterations that are not necessary for our algorithm and the one in [9]. Moreover, at each iteration of the algorithm in [21], it is required to solve a single variable equation and the corresponding linear optimization problem for each grid node. Finally, we emphasize here that the efficiency of the algorithms (of [9, 21] and ours) strongly depends upon the number of grid nodes (or subproblems solved) that are associated with the dimension of the grid points, under the condition of the same input size and the tolerance *ε* value. In fact, the nonuniform grid in [9] derives from parting a *k*-dimensional hypercube. Therefore, from the procedure of the algorithm and its computational complexity analysis it can be seen that our work is independent of [9, 21] and the proposed algorithm differs significantly giving an interesting alternative approach to solve the problem with a reduced running time.

The structure of this paper is as follows. The next section describes the equivalent problem and its decomposition technique. Section 3 presents the algorithm and the computational cost of such an algorithm. Finally, some conclusions are drawn in Sections 4 and 5, and discussions presented.

## Equivalent problem and its decomposition technique

### Equivalent problem

For solving problem (P), we will propose an equivalent problem (P). To this end, let us firstly denote

2.1 $$ l_{i}=\min_{x\in\Omega}a^{\top}_{i}x,\qquad u_{i}=\max_{x\in\Omega}a^{\top}_{i}x,\quad i=1,\ldots,k. $$

Assume that, without loss of generality, $k= \arg\max\{\frac {u_{i}}{l_{i}} | i=1,\ldots,k\}$, and define a rectangle *H* given by

2.2 $$ H=[l_{1},u_{1}] \times[l_{2},u_{2}]\times\cdots\times[l_{k-1},u_{k-1}]. $$

Thus, by introducing variable $y\in R^{k-1}$, problem (P) is equivalent to the following problem:

$$\text{(Q)}:\quad \textstyle\begin{cases} \text{min } \varphi(y_{1},\ldots,y_{k-1},a^{\top}_{k}x)\\ \text{s.t. }a^{\top}_{i}x\leqslant y_{i}, i=1,\ldots,k-1,\\ \hphantom{\text{s.t. }}x\in\Omega,\\ \hphantom{\text{s.t. }}y=(y_{1},\ldots,y_{k-1})\in H. \end{cases} $$

The key equivalent theorem for problems (P) and (Q) is given as follows.

#### Theorem 1

$x^{\ast}\in R^{n}$ *is a global optimum solution of problem* (*P*) *if and only if* $(x^{\ast},y^{\ast})\in R^{n+k-1}$ *is a global optimum solution of problem* (*Q*), *where* $y_{i}^{\ast}=a^{\top }_{i}x^{\ast}$ *for each* $i=1,\ldots,k-1$. *In addition*, *the global optimal values of problems* (*P*) *and* (*Q*) *are equal*.

#### Proof

If $x^{\ast}$ is a global optimal solution of problem (P), let

$$y_{i}^{\ast}=a^{\top}_{i}x^{\ast},\quad i=1, \ldots,k-1. $$

It is obvious that $(x^{\ast},y^{\ast})\in R^{n+k-1}$ is a feasible solution of problem (Q). Let $(x,y)$ be any feasible solution of problem (Q), i.e.,

2.3 $$ a^{\top}_{i}x\leqslant y_{i},\quad i=1, \ldots,k-1, x\in\Omega. $$

According to the definition of $y^{\ast}$ and the optimality of $x^{\ast }$, we must have

2.4 $$ \varphi\bigl(y^{\ast}_{1},\ldots,y^{\ast}_{k-1},a^{\top}_{k}x^{\ast} \bigr) =\varphi\bigl(a^{\top}_{1}x^{\ast}, \ldots,a^{\top}_{k-1}x^{\ast},a^{\top }_{k}x^{\ast} \bigr) \leqslant\varphi\bigl(a^{\top}_{1}x,\ldots,a^{\top}_{k-1}x,a^{\top}_{k}x \bigr). $$

Additionally, from (2.3) and the assumption ($\mathbb{A}1$), it follows that

2.5 $$ \varphi\bigl(a^{\top}_{1}x,\ldots,a^{\top}_{k-1}x,a^{\top}_{k}x \bigr) \leqslant\varphi\bigl(y_{1},\ldots,y_{k-1},a^{\top}_{k}x \bigr). $$

Thus, (2.4) and (2.5) mean that $(x^{\ast},y^{\ast})$ is a global optimal solution to problem (Q).

Conversely, suppose that $(x^{\ast},y^{\ast})$ is a global optimal solution for problem (Q), then we have

$$a^{\top}_{i}x^{\ast}\leqslant y^{\ast}_{i},\quad i=1,\ldots,k-1, x^{\ast }\in\Omega. $$

By the assumption of *φ*, we can obtain

$$\varphi\bigl(a^{\top}_{1}x^{\ast},\ldots,a^{\top}_{k-1}x^{\ast},a^{\top }_{k}x^{\ast} \bigr) \leqslant\varphi\bigl(y^{\ast}_{1},\ldots,y^{\ast}_{k-1},a^{\top}_{k}x^{\ast} \bigr). $$

For any given $x\in\Omega$, if we let $y_{i}=a^{\top}_{i}x, i=1,\ldots ,k-1$, then $(x,y)$ is a feasible solution to problem (Q) with $y=(y_{1},\ldots,y_{k-1})\in R^{k-1}$. Thus, from the optimality of $(x^{\ast},y^{\ast})$ it follows that

$$\begin{aligned} \varphi\bigl(a^{\top}_{1}x^{\ast},\ldots,a^{\top}_{k-1}x^{\ast},a^{\top }_{k}x^{\ast} \bigr) &\leqslant\varphi\bigl(y^{\ast}_{1},\ldots,y^{\ast}_{k-1},a^{\top}_{k}x^{\ast} \bigr) \\ & \leqslant\varphi\bigl(y_{1},\ldots,y_{k-1},a^{\top}_{k}x \bigr) =\varphi\bigl(a^{\top}_{1}x,\ldots,a^{\top}_{k-1}x,a^{\top}_{k}x \bigr). \end{aligned} $$

This means that $x^{\ast}$ is a global optimal solution to problem (P). □

By Theorem 1, we can conclude that, for solving the problem (P), we may globally solving its equivalent problem (Q) instead. Besides, it is easy to understand that the problems (P) and (Q) have the same global optimal value. Hence, we will propose a decomposition approach for the problem (Q) below.

### Linear decomposition technique

Problem (Q) has a relatively low-rank decomposition structure because, in contrast to problem (P), the nonconvexity of the objective function only involves the term $a^{\top}_{k}x$ if we fix a $y=(y_{1},\ldots ,y_{k-1})\in H$. In order to solve problem (Q), based on this observation, for any given $\theta\in(0,1)$ we want to construct a polynomial size grid by subdividing *H* into smaller rectangles, such that the ratio of successive divisions is equal to $(1+\theta)$ in each dimension. Thus, a polynomial size grid will be generated over *H*, where the set of the grid nodes can be given by

2.6 $$ B^{\theta}=\bigl\{ (\upsilon_{1}, \upsilon_{2},\ldots,\upsilon_{k-1}) | \upsilon_{i} \in\mathcal{D}^{\theta}_{i}, i=1,\ldots,k-1\bigr\} , $$

where $\mathcal{D}^{\theta}_{i}=\{l_{i}, l_{i}(1+\theta), \ldots, l_{i}(1+\theta)^{r_{i}}\}$ with

2.7 $$ r_{i}=\arg\max\bigl\{ \tau\in\mathbb{N} | l_{i}(1+\theta)^{\tau}\leq u_{i}\bigr\} ,\quad i=1, \ldots,k-1. $$

Note that under the assumption ($\mathbb{A}3$), $l_{i}>0$ must hold for each *i*. Clearly, for any $(y_{1},y_{2},\ldots, y_{k-1})\in H$, there exists a point $(\upsilon_{1},\upsilon_{2},\ldots,\upsilon _{k-1})\in B^{\theta}$ such that

$$y_{i}\in\bigl[\upsilon_{i},(1+\theta)\upsilon_{i} \bigr]\quad \text{for each } i=1,\ldots,k-1. $$

Thus, *H* can be approximated by the set $B^{\theta}$. Next, for each grid node $\upsilon\in B^{\theta}$, consider the corresponding subproblem as follows:

$$\mathrm{P1}(\upsilon):\quad \textstyle\begin{cases} \omega(\upsilon)=\text{min } \varphi(\upsilon,a^{\top}_{k}x),\\ \hphantom{\omega(\upsilon)={}}\text{s.t. } a^{\top}_{i}x\leqslant\upsilon _{i}, i=1,\ldots,k-1,\\ \hphantom{\omega(\upsilon)={}\text{s.t. }}x\in\Omega. \end{cases} $$

Notice that, by the assumption ($\mathbb{A}1$) of *φ*, for a given $\upsilon\in B^{\theta}$, problem $\mathrm{P1}(\upsilon)$ is equivalent to a linear problem $\mathrm{P2}(\upsilon)$:

$$\mathrm{P2}(\upsilon):\quad \textstyle\begin{cases} \text{min } a^{\top}_{k}x,\\ \text{s.t. } a^{\top}_{i}x\leqslant\upsilon_{i}, i=1,\ldots,k-1,\\ \hphantom{\text{s.t. }}x\in\Omega. \end{cases} $$

That is, for a fixed point $\upsilon\in B^{\theta}$, $x^{\upsilon}$ is the optimal solution of problem $\mathrm{P1}(\upsilon)$ if and only if $x^{\upsilon}$ is an optimal solution for problem $\mathrm{P2}(\upsilon)$.

Clearly, for each $\upsilon\in B^{\theta}$, the corresponding subproblems $\mathrm{P1}(\upsilon)$ can easily be solved by a linear program $\mathrm{P2}(\upsilon)$. Thus, we can decompose a nonconvex programming problem (Q) into a series of subproblems, and we can obtain its approximation global solution via the solutions of those linear programming problems when concerning all nodes *υ* over $B^{\theta}$.

## Algorithm and its computational complexity

In this section, we will propose an effective algorithm for getting the approximation solution to problem (P), and then analyze its computational complexity.

### *ε*-approximation algorithm

In what follows we will introduce an algorithm for solving problem (P), and the algorithm is able to return an *ε*-approximate solution of problem (P).

Based on the particularities of problem (P), a given rectangle *H* is firstly subdivided to construct a necessary nonuniform grid $B^{\theta }$. The prime problem (P) can then transformed and decomposed into a series of subproblems on the basis of the exploration of the grid nodes. Each subproblem is associated with a grid node in the proposed algorithm, and it can be solved by a linear program. An necessary and specific description is given as follows. Given $\varepsilon\in(0,1)$, let $\theta=(1+\varepsilon)^{\frac {1}{c}}-1$. The grid nodes set $B^{\theta}$ can be generated by (2.6)-(2.7). For each $\upsilon\in B^{\theta}$, solve problem $\mathrm{P2}(\upsilon)$ to get the solution $x^{\upsilon}$, and the optimal value to the corresponding problem $\mathrm{P1}(\upsilon)$ is denoted $\omega(\upsilon)=\varphi(\upsilon,a^{\top}_{k}x^{\upsilon})$, here, let $\omega({\upsilon})= +\infty$ if the feasible set to $\mathrm{P2}(\upsilon)$ is empty. The process is repeated until all the points of $B^{\theta}$ are considered. The detailed algorithm is Algorithm 1.

**Algorithm 1:**
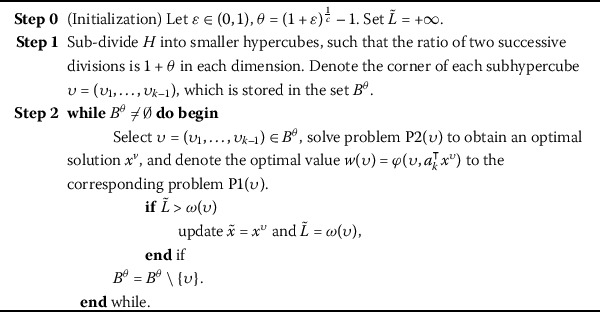
Algorithm statement

The following theorem shows that the proposed algorithm can reach an optimal solution to problem (P).

#### Theorem 2

*Given* $\varepsilon>0$, *an* *ε*-*optimal solution* *x̃* *to problem* (P) *from the proposed algorithm can be obtained in the sense that*

$$f(\tilde{x})\leq(1+\varepsilon)f\bigl(x^{\ast}\bigr), $$

*where* $x^{\ast}$ *is the optimal solution of problem* (P).

#### Proof

Let

3.1 $$ y_{i}^{\ast}=a^{\top}_{i}x^{\ast},\quad i=1, \ldots,k-1. $$

From $x^{\ast}$ being the optimal solution of problem (P), we have

$$l_{i}\leq y^{\ast}_{i}=a^{\top}_{i}x^{\ast} \leq u_{i},\quad i=1,\ldots,k-1. $$

This implies that $(y^{\ast}_{1},y^{\ast}_{2},\ldots,y^{\ast}_{k-1})\in H$, so there exists some $\upsilon^{\ast}\in B^{\theta}$ which satisfies

3.2 $$ (1+\theta)^{-1}\upsilon^{\ast}_{i}\leq y^{\ast}_{i}\leq\upsilon^{\ast }_{i},\quad i=1, \ldots,k-1. $$

Thus, combining with the assumptions of *φ*, we have

3.3 $$ f\bigl(x^{\ast}\bigr)=\varphi\bigl(y_{1}^{\ast}, \ldots,y_{k-1}^{\ast},a^{\top }_{k}x^{\ast} \bigr)\geq(1+\theta)^{-c}\varphi\bigl(\upsilon^{\ast},(1+\theta )a^{\top}_{k}x^{\ast}\bigr) \geq(1+\theta)^{-c} \varphi\bigl(\upsilon^{\ast},a^{\top}_{k}x^{\ast} \bigr). $$

Now, suppose that *x̄* is the optimal solution of problem $\mathrm{P1}(v^{\ast})$. Then $x^{\ast}\in\Omega$ together with (3.1)-(3.2) implies that $x^{\ast}$ is a feasible solution of problem $\mathrm{P1}(v^{\ast})$. Thus we have

3.4 $$ \varphi\bigl(\upsilon^{\ast},a^{\top}_{k}x^{\ast} \bigr)\geq\varphi\bigl(\upsilon^{\ast },a^{\top}_{k} \bar{x}\bigr). $$

Additionally, let $\tilde{\upsilon}=\arg\min\{\omega(\upsilon) | \upsilon\in B^{\theta}\}$. Since *x̃* is the optimal solution of problem $\mathrm{P1}(\tilde{\upsilon})$, it follows that $a_{i}^{\top}\tilde {x}\leq\tilde{v}_{i}, i=1,\ldots,k-1$, thus, we can get

3.5 $$ \varphi\bigl(\tilde{\upsilon},a_{k}^{\top} \tilde{x}\bigr)\geq \varphi\bigl(a^{\top}_{1} \tilde{x},a^{\top}_{2}\tilde{x},\ldots,a^{\top }_{k} \tilde{x}\bigr)=f(\tilde{x}). $$

According to the definitions of *ṽ* and *x̄*, we have

3.6 $$ \varphi\bigl(\upsilon^{\ast},a_{k}^{\top} \bar{x}\bigr)\geq\varphi\bigl(\tilde{\upsilon },a_{k}^{\top} \tilde{x}\bigr). $$

Hence, from (3.3)-(3.6) and $\theta=(1+\varepsilon)^{\frac {1}{c}}-1$, we can conclude that

$$f(\tilde{x})\leq(1+\varepsilon)f\bigl(x^{\ast}\bigr), $$

and so *x̃* is the approximation solution to problem (P). □

By Theorem 1 we also have the following corollary.

According to the above discussion, the *ε*-approximation solution to problem (P) can be obtained by solving $\vert B^{\theta } \vert $ (the number of grid nodes in $B^{\theta}$) linear programming problems $\mathrm{P2}(\upsilon)$ with $\upsilon\in B^{\theta}$. However, it is not necessary to solve each $\mathrm{P2}(\upsilon)$ associated with each $\upsilon\in B^{\theta}$ for searching the solution of problem (P), that is, by using the following proposition we can obtain an improvement of the algorithm.

#### Proposition 1

*Let* $\hat{x}=\arg\min\{a^{\top}_{k}x | x\in\Omega\}$. *Then* *x̂* *is an optimal solution of problem P*1$(\upsilon)$ *for any* $\upsilon\in\hat{B}^{\theta}$, *where*

3.7 $$ \hat{B}^{\theta}=\bigl\{ \upsilon\in B^{\theta} | a^{\top}_{i}\hat{x}\leq \upsilon_{i},i=1,\ldots,k-1 \bigr\} . $$

#### Proof

Suppose that $\bar{x}^{\upsilon}$ is any feasible solution of problem $\mathrm{P1}(\upsilon)$ with $\upsilon\in\hat{B}^{\theta}$. By using the definition of *x̂* we can see that *x̂* is a feasible solution of problem P1$(\upsilon)$ for any $\upsilon\in\hat{B}^{\theta }$. With the increase of the function *φ*, it follows that

$$\varphi\bigl(\upsilon,a^{\top}_{k}\hat{x}\bigr)\leq\varphi \bigl(\upsilon,a^{\top }_{k}\bar{x}^{\upsilon}\bigr),\quad \forall \upsilon\in\hat{B}^{\theta}, $$

which concludes the proof. □

Proposition 1 shows that *x̂* is the optimal solution of subproblem $\mathrm{P1}(\upsilon)$ for any $\upsilon\in\hat{B}^{\theta}$. Therefore, in practical implementations, we only are required to solve the subproblem $\mathrm{P2}(\upsilon)$ associated with the points contained in the set $B^{\theta} \setminus \hat{B}^{\theta}$. A further note on $\hat {B}^{\theta}$ is as follows.

For any $\theta\in(0,1)$, by the definition of *H*, let

3.8 $$ q_{i}=\arg\min\bigl\{ p\in\mathbb{N} | \hat{y}_{i}\leq l_{i}(1+\theta )^{p}\leq u_{i}\bigr\} \quad\text{for } i=1,\ldots,k-1, $$

where $\hat{y}_{i}=a^{\top}_{i}\hat{x}$ with $\hat{x}=\arg\min\{a^{\top }_{k}x | x\in\Omega\}$. Combining the definition of $r_{i},i=1,\ldots,k-1$ with the above result, the set $\hat{B}^{\theta}$ can be given by

3.9 $$ \hat{B}^{\theta}=\bigl\{ \bigl(l_{1}(1+ \theta)^{\sigma _{1}},\ldots ,l_{k-1}(1+\theta)^{\sigma_{k-1}}\bigr) | \sigma_{i}\in\{ q_{i},\ldots ,r_{i}\},i=1, \ldots,k-1\bigr\} . $$

Let

3.10 $$ T^{\theta}=B^{\theta} \setminus \hat{B}^{\theta}. $$

This means the *ε*-approximation solution to problem (P) can be obtained only by solving $\vert T^{\theta} \vert $ (the number of points in the set $T^{\theta}$) linear programming subproblems $\mathrm{P2}(\upsilon)$ for all $\upsilon\in T^{\theta}$. Thus the proposed algorithm can be improved by Algorithm 2.

**Algorithm 2:**
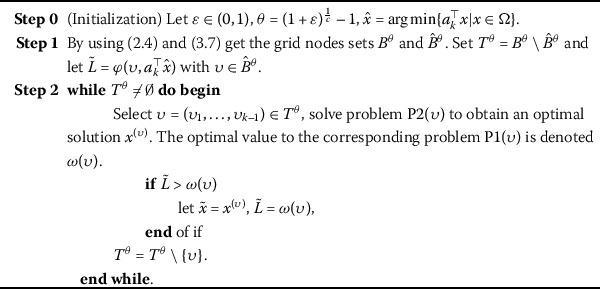
The improved algorithm

Notice that, when the proposed improved algorithm stops, we can obtain an *ε*-optimal solution *x̃* to problem (P) with the objective value *L̃*.

### Computational complexity for the algorithm

Now we consider the complexity analysis of the proposed improved algorithm. By (3.8)-(3.10), we can conclude that the number of the grid nodes belonging to $\hat{B}^{\theta}$ is at least

3.11 $$ \prod_{i=1}^{k-1} \log_{(1+\theta)} \biggl(\frac{u_{i}}{\hat{y}_{i}} \biggr), $$

where $\hat{y}_{i}=a^{\top}_{i}\hat{x}$ with $\hat{x}=\arg\min\{a^{\top }_{k}x | x\in\Omega\}$. On the other hand, we know from (2.4) that the total number of the points in the set $B^{\theta}$ is equal to $\prod_{i=1}^{k-1}r_{i}$, $r_{i}$ satisfying (2.5). Thus, it follows that the number of the elements in $B^{\theta}$ is at most

3.12 $$ \prod_{i=1}^{k-1} \log_{(1+\theta)} \biggl(\frac{u_{i}}{l_{i}} \biggr). $$

Combining (3.9) with (3.10), the proposed improvement algorithm requires that the number of the grid nodes considered in actually computation is not more than

3.13 $$ \Xi=\prod_{i=1}^{k-1} \biggl( \log_{(1+\theta)}\frac{u_{i}}{l_{i}} \biggr) -\prod_{i=1}^{k-1} \biggl(\log_{(1+\theta)}\frac{u_{i}}{\hat{y}_{i}} \biggr). $$

#### Theorem 3

*Let* $\hat{x}=\arg\min\{a^{\top}_{k}x | x\in \Omega\}$, $L=\min_{i=1,\ldots,k-1}\{\frac{u_{i}}{\hat{y}_{i}}\}$ *with* $\hat{y}_{i}=a^{\top}_{i}\hat{x}$, *and let* *U*=$\max_{i=1,\ldots,k-1}\frac{u_{i}}{l_{i}}$. *When* *k* *is fixed*, *the running time of the improved algorithm for obtaining an* *ε*-*optimal solution for problem* (P), *is bounded from above by*

$$O \biggl(\log\frac{U}{L}\cdot\frac{c^{k-1}\xi^{k-2} }{\varepsilon ^{k-1}}\cdot\operatorname{cost} \bigl( \vert \pi \vert ,n\bigr) \biggr), $$

*where* $\xi\in(\log L,\log U)$, *and* $\operatorname{cost}( \vert \pi \vert ,n)$ *is the time taken to solve a linear program in* *n* *variables and input size of* $\vert \pi \vert $ *bits*.

#### Proof

By Step 0 of the improved algorithm, it follows that

$$ \log_{(1+\theta)} \biggl(\frac{u_{i}}{\hat{y}_{i}} \biggr)\geq\log _{(1+\theta)}L= \frac{c\log L}{\log(1+\varepsilon)} $$

and

$$ \log_{(1+\theta)} \biggl(\frac{u_{i}}{l_{i}} \biggr)=\frac{\log(\frac {u_{i}}{l_{i}})}{\log(1+\theta)} = \frac{c\log(\frac{u_{i}}{l_{i}})}{\log(1+\varepsilon)}\leq\frac{c\log U}{\log(1+\varepsilon)}. $$

From the above results and (3.13), we have

3.14 $$ \begin{aligned}[b] \Xi &\leq \biggl(\frac{c\log U}{\log(1+\varepsilon)} \biggr)^{k-1}- \biggl( \frac{c\log L}{\log(1+\varepsilon)} \biggr)^{k-1} \\ &=\frac{c^{k-1}}{[\log(1+\varepsilon)]^{k-1}} \bigl[(\log U)^{k-1} -(\log L)^{k-1} \bigr]. \end{aligned} $$

Thus, the upper bound of the number of grid points Ξ is

3.15 $$ \frac{c^{k-1}}{\varepsilon^{k-1}} \bigl[(\log U)^{k-1} -(\log L)^{k-1} \bigr]. $$

The result of (3.15) holds because $\log(1+\varepsilon)\approx \varepsilon$ for small *ε* values. By using the Lagrange mean value theorem, there exists some $\xi\in (\log L,\log U)$ such that

3.16 $$ (\log U)^{k-1}-(\log L)^{k-1}=(k-1) \xi^{k-2}(\log U-\log L). $$

Thus we can know from (3.14)-(3.16) that the total number of the grid nodes considered in the improved algorithm is not more than

$$\frac{(k-1)\xi^{k-2}c^{k-1}(\log U-\log L)}{\varepsilon^{k-1}}. $$

Note that log*U* and log*L* are computed in polynomial time about the input size of the problem. Additionally, for each grid node *υ* in the set $T^{\theta}$, a corresponding linear programming problem $\mathrm{P2}(\upsilon)$ is required to solve. Therefore, for a fixed *k*, the running time required by the improved algorithm for obtaining an *ε*-optimal solution for problem (P), is bounded from above by

3.17 $$ O \biggl(\log\frac{U}{L}\cdot\frac{c^{k-1}\xi ^{k-2} }{\varepsilon^{k-1}}\cdot \operatorname{cost}\bigl( \vert \pi \vert ,n\bigr) \biggr), $$

where $\xi\in(\log L,\log U)$. □

In view of the above theorem we can conclude that the running time of the proposed improved algorithm is polynomial in input size and $\frac {1}{\varepsilon}$ for fixed *k*, hence the algorithm is an FPTAS (fully polynomial-time approximation scheme) for the problem (P).

*Comparison with* [9, 21]*:* The algorithm in [9] searches for the optimal objective value in a *k*-dimensional grid, in which requires one to check the feasible of a linear program for each grid node, thus the total number of linear programs solved by their method is $O(\frac{c^{k}(\log\frac {M}{m})^{k}}{\varepsilon^{k}})$ with $M\setminus m=\max \setminus \min\{ a_{i}^{\top}x | x\in\Omega, i=1,\ldots,k\}$. In the algorithm [21], the number of linear optimization problems that are solved over a convex set in each iteration is $O(\frac{c^{k-1}(\log R)^{k-1}}{\varepsilon^{k-1}})$, where $R=\max\{ \frac{u_{i}}{l_{i}}|i=1,\ldots,k\}$. Also, at each iteration of their algorithm [21], the ratio of the upper and lower bounds of the objective value can be reduced by a constant factor, hence the number of iterations is $O(\frac{c}{\varepsilon}\cdot\log\frac{z^{0}_{U}}{z^{0}_{L}})$, where $z^{0}_{U}(z^{0}_{L})$ denotes the initial upper (lower) bound on the objective value. This implies that the algorithm in [21] solves $O(\log\frac{z^{0}_{U}}{z^{0}_{L}}\cdot\frac{c^{k}(\log R)^{k-1}}{\varepsilon^{k}})$ linear optimization problems over a convex set. In this article, as can be seen in (3.17), the proposed algorithm solves $O (\log\frac{U}{L}\cdot\frac{c^{k-1}\xi^{k-2} }{\varepsilon ^{k-1}} )$ different linear programs, and the running time is associated with $(k-1)$th order in $\frac{1}{\varepsilon}$, compared with the *k*th order in $\frac{1}{\varepsilon}$ in [9, 21].

## Conclusions

In this article, we present a new linear decomposition algorithm for globally solving a class of nonconvex programming problems. First, the original problem is transformed and decomposed into a polynomial number of equivalent linear programming subproblems, by exploiting a suitable nonuniform grid. Second, compared with existing results in the literature, the proposed algorithm does not require the assumptions of quasi-concavity and differentiability of the objective function, and further, the rank *k* of the objective function is not limited to only around two. Finally, the computational complexity of the algorithm is given to show that it differs significantly giving an interesting alternative approach to solve the problem (P) with a reduced running time.

## Results and discussion

In this work, a new linear decomposition algorithm for globally solving a class of nonconvex programming problems is presented. As further work, we think the ideas can be extended to more general type optimization problems, in which each $a^{\top}_{i}x$ in the objective function to problem (P) is replaced with a convex function.
